# 1018. Outcomes in Intravenous to Oral Antimicrobial Therapy in Uncomplicated Beta-hemolytic Streptococcus Bloodstream Infections in Immunocompromised Individuals

**DOI:** 10.1093/ofid/ofad500.049

**Published:** 2023-11-27

**Authors:** Mackenzie R Keintz, Cristina J Torres, Molly M Miller, Bryan T Alexander, Elizabeth Lyden, Jihyun Ma, Erica J Stohs, Trevor C Van Schooneveld, Jasmine R Marcelin

**Affiliations:** University of Nebraska Medical Center, Omaha, NE; University of Nebraska Medical Center, Omaha, NE; Nebraska Medicine, Omaha, Nebraska; Nebraska Medicine, Omaha, Nebraska; University of Nebraska Medical Center, Omaha, NE; University of Nebraska Medical Center, Omaha, NE; University of Nebraska Medical Center, Omaha, NE; University of Nebraska Medical Center, Omaha, NE; University of Nebraska Medical Center, Omaha, NE

## Abstract

**Background:**

β-hemolytic streptococci commonly cause bloodstream infections (BSI) in immunocompromised individuals, who may receive long courses of intravenous (IV) antibiotics. Accumulating evidence reveal that shorter courses of oral antibiotic therapy (OAT) may be an acceptable alternative, but immunocompromised hosts are frequently excluded from these studies. We evaluated outcomes in IV to OAT transitions for treating β-hemolytic streptococcal uncomplicated BSI (uBSI) in immunocompromised hosts.

**Methods:**

This retrospective cohort study evaluated immunocompromised adult patients hospitalized between 1/1/2013 and 12/31/2019 with uBSI due to β-hemolytic Streptococcus. Patients were excluded if source of infection was endovascular, central nervous system or bone/joint infection without definitive surgical intervention. We compared outcomes between IV only vs OAT cohorts. Primary outcome was 30-day all-cause mortality. Other outcomes included length of stay (LOS), BSI relapse, 30-day rehospitalization, and adverse drug events. The primary outcome was compared to previously presented findings in immunocompetent patients. Comparisons between groups were performed using Fisher’s exact test or Mann-Whitney test.

**Results:**

Of 321 BSI screened, 52 immunocompromised adults with β-hemolytic Streptococcus uBSI identified. OAT was used in 25 episodes (48%). Cohort demographics were similar. PITT bacteremia score ≥4 was higher in the IV only cohort (30% vs. 0% p< 0.05). Immunocompromised categories primarily included patients with solid organ transplant/hematopoietic marrow transplant (44%) and heme/solid malignancy on chemotherapy (48%). Thirty-day mortality was lower in the OAT cohort (8% vs. 33% p< 0.05). There was no difference in LOS, readmission, BSI relapse or adverse events between cohorts (Table 1). There was no difference in 30-day mortality in OAT between immunocompromised and immunocompetent individuals (8% vs 2% p=0.15) despite overall increased 30-day mortality in immunocompromised patients (Table 2).

Comparison of demographics and outcomes between OAT and IV only cohorts

**Figure:**
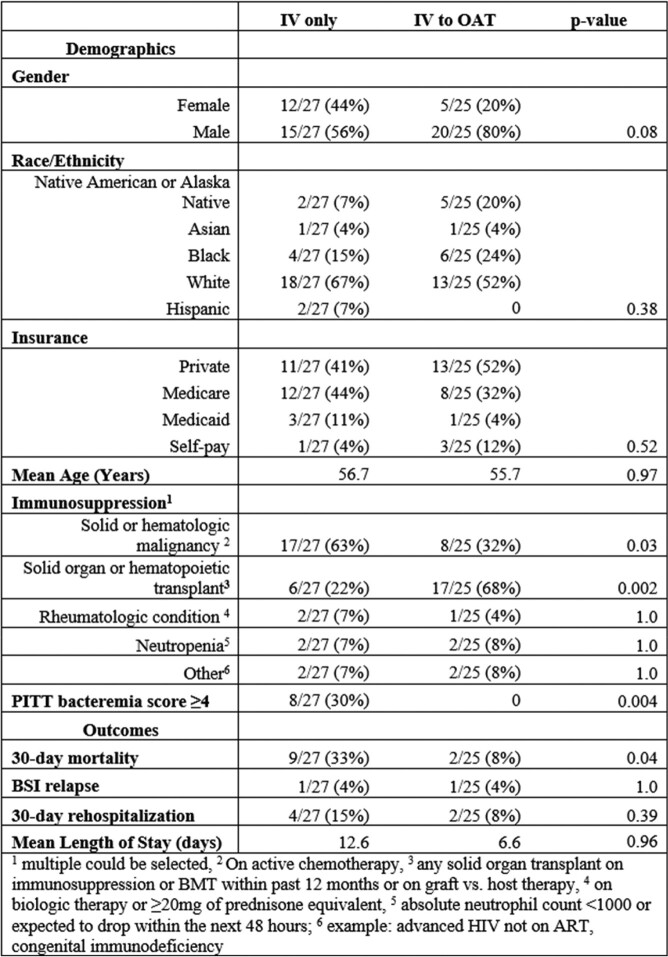

Comparison of outcomes between immunocompromised and immunocompetent individuals

**Figure:**
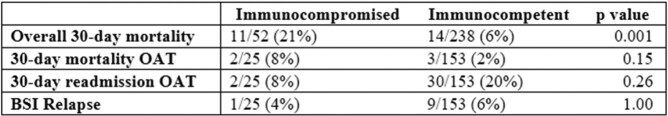

**Conclusion:**

Opportunities exist to modify management of uBSI in immunocompromised hosts. Transition to OAT did not increase 30-day mortality and was equivalent to immunocompetent hosts transitioned to OAT.

**Disclosures:**

**Bryan T. Alexander, PharmD, BCIDP, AAHIVP**, Astellas Pharma: Advisor/Consultant|F2G: Advisor/Consultant|Merck: Grant/Research Support **Erica J. Stohs, MD, MPH**, bioMerieux: Grant/Research Support|Merck: Grant/Research Support **Trevor C. Van Schooneveld, MD, FSHEA, FACP**, AN2 Therapeutics: Grant/Research Support|Biomeriuex: Advisor/Consultant|Biomeriuex: Grant/Research Support|Insmed: Grant/Research Support|Thermo-Fischer: Honoraria

